# Molecular characterization of chikungunya virus during the 2019 outbreak in the Democratic Republic of the Congo

**DOI:** 10.1080/22221751.2020.1810135

**Published:** 2020-09-02

**Authors:** Philippe Selhorst, Sheila Makiala-Mandanda, Birgit De Smet, Joachim Mariën, Colin Anthony, Guillaume Binene-Mbuka, Anja De Weggheleire, Gillon Ilombe, Eddy Kinganda-Lusamaki, Elisabeth Pukuta-Simbu, Leopold Lubula, Placide Mbala-Kingebeni, Antoine Nkuba-Ndaye, Florian Vogt, Francis Watsenga, Wim Van Bortel, Veerle Vanlerberghe, Kevin K. Ariën, Steve Ahuka-Mundeke

**Affiliations:** a Department of Biomedical Sciences, Institute of Tropical Medicine, Antwerp, Belgium; b The Outbreak Research Team, Institute of Tropical Medicine, Antwerp, Belgium; c Department of Virology, Institut National de Recherche Biomédicale, Kinshasa, Democratic Republic of the Congo; d University of Kinshasa, Kinshasa, Democratic Republic of the Congo; e Department of Clinical Sciences, Institute of Tropical Medicine, Antwerp, Belgium; f Department of Pathology, Institute of Infectious Disease, University of Cape Town, Cape Town, South Africa; g Department of Entomology, Institut National de Recherche Biomédicale, Kinshasa, Democratic Republic of the Congo; h University of Antwerp, Antwerp, Belgium; i Direction Generale de Lutte contre la Maladie (DGLM), Kinshasa, Democratic Republic of the Congo; j Department of Public Health, Institute of Tropical Medicine, Antwerp, Belgium

**Keywords:** Chikungunya, CHIKV, A226V, *Aedes albopictus*, Democratic Republic of the Congo, DRC, outbreak, I211T

## Abstract

Early 2019, a chikungunya virus (CHIKV) outbreak hit the Democratic Republic of the Congo (DRC). Though seldomly deadly, this mosquito-borne disease presents as an acute febrile (poly)arthralgia often followed by long-term sequelae. Although *Aedes aegypti* is the primary vector, an amino acid substitution in the viral envelope gene E1 (A226V) is causing concern as it results in increased transmission by *Aedes albopictus*, a mosquito with a much wider geographical distribution. Between January and March 2019, we collected human and mosquito samples in Kinshasa and Kongo Central province (Kasangulu and Matadi). Of the patients that were tested within 7 days of symptom onset, 49.7% (87/175) were RT–qPCR positive, while in the mosquito samples CHIKV was found in 1/2 pools in Kinshasa, 5/6 pools in Kasangulu, and 8/26 pools in Matadi. Phylogenetic analysis on whole-genome sequences showed that the circulating strain formed a monophyletic group within the ECSA2 lineage and harboured the A226V mutation. Our sequences did not cluster with sequences from previously reported outbreaks in the DRC nor with other known A226V-containing ECSA2 strains. This indicates a scenario of convergent evolution where A226V was acquired independently in response to a similar selection pressure for transmission by *Ae. albopictus*. This is in line with our entomological data where we detected *Ae. albopictu*s more frequently than *Ae. aegypti* in two out of three affected areas. In conclusion, our findings suggest that CHIKV is adapting to the increased presence of *Aedes albopictus* in DRC.

## Introduction

Chikungunya virus (CHIKV) is a mosquito-borne alphavirus belonging to the *Togaviridae* which causes an acute febrile (poly)arthralgia in humans. Other common acute symptoms include headache, myalgia, joint pain, fatigue, and maculopapular rash. Although seldomly deadly, acute infection is often followed by chronic discomfort (rheumatic pain, fatigue, and depression) which can last for several years [1].

CHIKV was initially discovered in Tanzania in 1953 and was first reported in Asia in 1958. Up until 2004, sporadic cases as well as larger outbreaks in the 1960s and 1990s were mainly confined to Africa and Asia with inter-epidemic periods ranging from seven to 20 years [2]. However, in 2004 the disease reappeared in Kenya spreading across the Indian Ocean islands and India to Southeast Asia infecting millions of people in large outbreaks with high attack rates. Since then CHIKV has established itself as a global pathogen, spreading throughout the Americas since 2013 and into Southern Europe [3].

CHIKV has a single-stranded, positive sense RNA genome of 12 kb consisting of four non-structural (nsP1, nsP2, nsP3, nsP4) and five structural proteins (C, E3, E2, 6 K, E1). Phylogenetic analysis has revealed four major lineages of CHIKV: the Asian (AS), West African (WA), East/Central/South African (ECSA), and Indian Ocean (IO) lineage [4]. The ECSA lineage can further be divided into two clades: ECSA1, entirely consisting of ancestral CHIKV sequences and ECSA2, which contains sequences from Central African Republic, Cameroon, Gabon, and Republic of the Congo (ROC) [4].

In Africa, CHIKV is maintained in a sylvatic cycle involving forest-dwelling mosquitoes and non-human primates, with urban penetration and human-to-human transmission being fuelled by two anthropophilic mosquitoes of the genus *Aedes: Ae. Aegypti*, an urban mosquito associated with most reported CHIKV transmissions worldwide, and *Ae. albopictus,* originally a zoophilic forest-dwelling mosquito species from Asia with a currently wider geographical distribution than *Ae. aegypti* [5]*.* Over the last decades, *Ae. albopictus* has become an increasingly more important vector of CHIKV. This has been linked to an amino acid substitution in the viral envelope gene E1 (A226V) which was first reported in the ECSA lineage in 2005. A226V was shown to increase midgut infection of *Ae*. *albopictus* (but not of *Ae. aegypti*) resulting in enhanced transmissibility of the virus by this vector species [6]. Since then other adaptive mutations to *Ae. albopictus* have been identified in E1 (A98T, K211E) [7] as well as in the glycoprotein E2 (D60G, R198Q, L210Q, I211T, K233E, K252Q) [8] which forms a heterodimer on the viral surface with E1. Similarly to E1-A226V, E2-L210Q is responsible for increased CHIKV dissemination in *Ae. albopictus* by increasing the initial infectivity for midgut epithelial cells [9]. E2-I211T on the other hand, has a low prevalence in ECSA viruses, yet it occurs in all viruses harbouring E1-A226V [10]. *In vitro* studies showed that E2-I211T works epistatically by providing a prerequisite background for E1-A226V to exert its effect on infectivity [10].

Here we present the phylogenetic characterization of CHIKV detected in human and mosquito samples from an outbreak in the Democratic Republic of the Congo (DRC) that most likely started late 2018 in Mont-Ngafula located in the southern part of the DRC's capital Kinshasa (personal communication with INRB). Previous outbreaks in Central Africa have been reported in Uganda (1961/1968), Angola (1962/1970), Equatorial Guinea (2002–2006), Central African Republic (1978/1984/2000–2003), Cameroon (2006), Gabon (2006–2012), and ROC (2011) [11]. In the DRC, CHIKV outbreaks were first reported in 1958 in Haut-Uéle province, north-eastern DRC, and subsequently in urban Kinshasa in 1999–2000 [12] and in 2012 [13]. However, due to the lack of a specific surveillance system, these reports provide an incomplete picture of CHIKV in the DRC and continuous CHIKV transmission has been shown to occur in between reported outbreaks [14]. This highlights the importance of the phylogenetic data presented here, adding to our understanding of CHIKV epidemiology in the DRC.

## Materials & methods

### Human sample collection

As part of the national efforts of outbreak investigation and response, blood samples and clinical data of patients presenting with chikungunya-like symptoms (i.e. recent and abrupt onset of fever and/or severe arthralgia) were collected by health care workers and sent to the “Institut National de Recherche Biomédicale” (INRB) in Kinshasa for diagnostic work-up. For this analysis, we used clinical data, laboratory testing results, and samples collected between January 7 to March 7 2019 in Kinshasa (health zones of Mont-Ngafula I and II), in Kasangulu (health zones of Masa), and Matadi. Blood collection and clinical evaluation were approved as a standard of care by the Ministry of Health of the DRC and oral consent was obtained from all patients before blood sampling.

### Mosquito collection and identification

Adult mosquitoes were sampled in the late afternoon during three days in Kinshasa, Kasangulu, and Matadi using two Prokopack aspirators in the vegetation surrounding houses with suspected human CHIKV cases. Water-holding containers located both indoors and outdoors, were inspected for mosquito larvae, which were subsequently reared to adulthood in the laboratory. Data were collected on standard entomological forms. Adult mosquitoes were killed by ethanol inhalation, identified as *Ae. aegypti* or *Ae. albopictus* based on morphological characteristics and pooled according to sampling site, sex, stage during capture (adult/larvae) and species. Mosquito pools were stored in Eppendorf tubes with RNAshield (Zymo research) at 4°C for a maximum of one week until transportation to INRB, where they were screened for the presence of CHIKV using the Zymo quick DNA/RNA pathogen extraction kit and RT–qPCR (described below). The CHIKV infection rates in pooled samples of the collected mosquitoes were estimated based on a maximum likelihood estimation using the Microsoft® Office Excel© Add-In package from the Centers for Disease Control and Prevention, U.S.A. [15].

### Lab-infected mosquitoes

To assess how many CHIKV positive mosquitoes would be needed to obtain a positive mosquito pool in our RT-qPCR and evaluate the variation in Ct-values between individual mosquitoes, lab-infected and negative *Ae. aegypti* mosquitoes were ordered from Infravec2 (SKU:V.1.1.5.I4.FR.4.19). Five to seven days post-emergence females were fed on an infectious blood-meal containing 10^7^ PFU/mL ECSA-CHIKV (Genbank DQ443544) using the Hemotek® system. Engorged females were then transferred to small containers and fed with 10% sucrose in a chamber maintained at 28°C ± 1°C, at 16h: 8 h light: dark cycle and 80% humidity. Mosquitoes were frozen at −80°C upon reaching disseminated infection seven days post-infection.

### RNA extraction and RT-qPCR on human and mosquito samples

RNA was extracted from human plasma samples (140 µL) using the QIAamp® Viral RNA Mini Kit (Qiagen, Hilden, Germany) as per manufacturer's instructions. Mosquitoes were pooled per 50 individuals or less and homogenized with a vortex in ZR bashing bead lysis tubes containing 1 mL DNA/RNA shield. RNA was subsequently extracted from 200 µL homogenate according to the protocol of the Quick-DNA/RNA^TM^ Pathogen Miniprep Kit (Zymo Research, Germany). RNA from phocine distemper virus (PDV) was added to all samples as an internal RNA extraction and PCR inhibition control [16]. A CHIKV-specific RT-qPCR was then performed with 5 µL RNA in a 25 µL reaction using the iTaq Universal Probes One-Step Kit from Bio-Rad by amplifying a 77 bp part of the nonstructural protein 1 (NSP-1) gene with primers and probes (S Table 1) detecting the African and Asian CHIKV strains as previously described [17]. Cycling conditions were 10 min at 50°C, a denaturation step of 5 min at 95°C, followed by 50 cycles of 10 s at 95°C and 30 s at 60°C. A PDV RT-qPCR was run in parallel (S table 1). RNA samples with *Ct*-value >30 were concentrated to 10 µL using the Zymo RNA Clean & Concentrator^TM^ 5 kit (Zymo Research) prior to sequencing.

### Whole genome sequencing using MinION

Samples with *Ct*-value <35 (*n* = 16) were selected for sequencing on an Oxford Nanopore MinION device using R9.4 flow cells (Oxford Nanopore Technologies, UK), based on a protocol from Quick et al., [18]. Sequencing statistics can be found in S Table 2. Briefly, extracted RNA from human or mosquito samples was converted to cDNA using random hexamers and the ProtoScript® II First Strand cDNA Synthesis Kit (New England Biolabs, UK). Subsequently, a strain-specific multiplex PCR was performed in four reactions using an ECSA primer scheme (S Table 3) and 35 cycles of PCR with Q5 High-Fidelity DNA polymerase (New England Biolabs). The resulting 800 bp PCR products were pooled and cleaned up using AmpureXP magnetic beads (Beckman Coulter, UK) and quantified using a Qubit dsDNA High Sensitivity assay on a Qubit 3.0 instrument (Thermo Fisher Scientific, USA). Samples were then barcoded using the Ultra II End Repair/dA-Tailing Module (New England Biolabs) and the native barcoding kits NBD104 and NBD114 (Oxford Nanopore Technologies), cleaned up with magnetic beads and pooled at equimolar ratios prior to ligation of the AMII adapters with blunt/TA ligase master mix (New England Biolabs). Sequencing libraries were loaded onto the R9.4 flow cell using the ligation sequencing kit LSK109 (Oxford Nanopore Technologies) and sequencing data were collected for 24–48 h. Sequence reads were basecalled using the Guppy algorithm in high accuracy mode (Oxford Nanopore Technologies) and demultiplexed using Porechop (https://github.com/rrwick/Porechop). Consensus genome sequences were produced by aligning to a CHIKV-ECSA reference genome (GenBank HQ456251.1) using Burrows–Wheeler Aligner (BWA-MEM). After removal of primer sequences using a custom Python script, a majority rule consensus was produced for positions with ≥100× genome coverage, while regions with lower coverage, were masked with N characters (https://github.com/ColinAnthony/nanopore_pipeline_wrapper).

### Maximum likelihood analysis

Sequences were aligned to a panel of previously published sequences (S Table 4) using MAFFT v7 and a maximum likelihood phylogenetic tree was inferred with IQ-TREE v1.6.12. Bayesian Information Criterion was used to select the General Time Reversible substitution model (GTR + F+I + G4) and 100 nonparametric bootstrap replicates. The tree was rooted using O’nyong-nyong virus as outgroup and visualized in iTOL v5.

## Results

Between January and March 2019, 175 blood samples were collected from suspect chikungunya fever cases with symptom onset since seven days or less, in Kinshasa (health zones of Mont-Ngafula I and II) and Kongo Central (Kasangulu and Matadi). About half (49.7%) were CHIKV RT-qPCR positive. The most frequent clinical symptoms of the confirmed cases were, arthralgia in mainly knees, wrist and/or ankles (93%), fever (90%), headache (56%), and asthenia (53%). Adult mosquitoes and larvae (reared to adulthood) were also captured in and around clusters of human cases in the affected localities. In Kasangulu and Matadi, most of the collected mosquitoes were *Ae. albopictus,* 99% (280/281) and 97% (1224/1258) respectively, but not in Kinshasa (32.5%, 28/86) (Table 1). CHIKV was found in one *Ae. aegypti* pool in Kinshasa, five *Ae. albopictus* pools in Kasangulu, and eight *Ae. albopictus* pools in Matadi. Interestingly, we found four male mosquito pools to be positive and, albeit at very high Ct values, we could also repeatedly detect CHIKV RNA in two larval pools (Table 1). To assess if a single positive mosquito can result in a positive pool in our RT-qPCR assay, we determined the Ct-values of 10 pools containing 49 negative mosquitoes and one positive mosquito. However, as we did not collect individual mosquitoes in the field, we had to perform this experiment with lab-infected mosquitoes. We observed a relatively low median Ct of 17.1 (range 14.5 – 29.6), which was not surprising considering these mosquitoes received roughly 100x more infectious bloodmeals as opposed to mosquitoes in nature [19]. Nevertheless, these results imply that one positive mosquito will result in a positive pool, and hence that the estimated infection rate of the pools varies between 0 and 4%.

**Table 1.** Information on mosquito pools collected during the outbreak investigation in Kinshasa, Matadi, and Kasangulu.

City	*Aedes* species	Sex	Stage at collection	Positive/ total pools	ML estimated infection rate [95% CI]	Mosquito number per pool	*Ct* values of positive pool(s)*
Kinshasa	*albopictus*	Unknown	adult	0/1	0 [0, 7.6]%	(20)	
			larvae	0/1	0 [0, 4.1]%	(38)	
	*aegypti*	Unknown	adult	1/1	Not possible	(6)	(16)
			larvae	0/1	0 [0, 6.9]%	(22)	
Matadi	*albopictus*	Male	adult	2/4	1.1 [2.2, 4.4]%	(50,50,50,50)	(37,20)
			larvae	2/5	0.9 [1.8, 3.5]%	(50,50,50,35,50)	(43,37)
		Female	adult	3/6	1.2 [3.4, 3.7]%	(50,50,50,50,50,50)	(29,16,17)
			larvae	0/6	0 [0, 1.1]%	(20, 50, 53, 50, 50, 50)	
		Unknown	adult	1/1	Not possible	(50)	(19)
			larvae	0/4	0 [0, 1.3]%	(50,50,50,50)	
Kasangulu	*albopictus*	Male	adult	2/2	Not possible	(50, 46)	(34, 21)
		Female	adult	2/2	Not possible	(55, 61)	(33, 18)
		Unknown	adult	0/1	0 [0, 4.3]%	(36)	
			larvae	1/1	Not possible	(38)	(39)

Note: *The order of the *Ct*-values corresponds to the order of the mosquito number per pool in red.

CI: confidence interval; ML: maximum-likelihood; *Ct*: cycle threshold.

**Figure 1.**
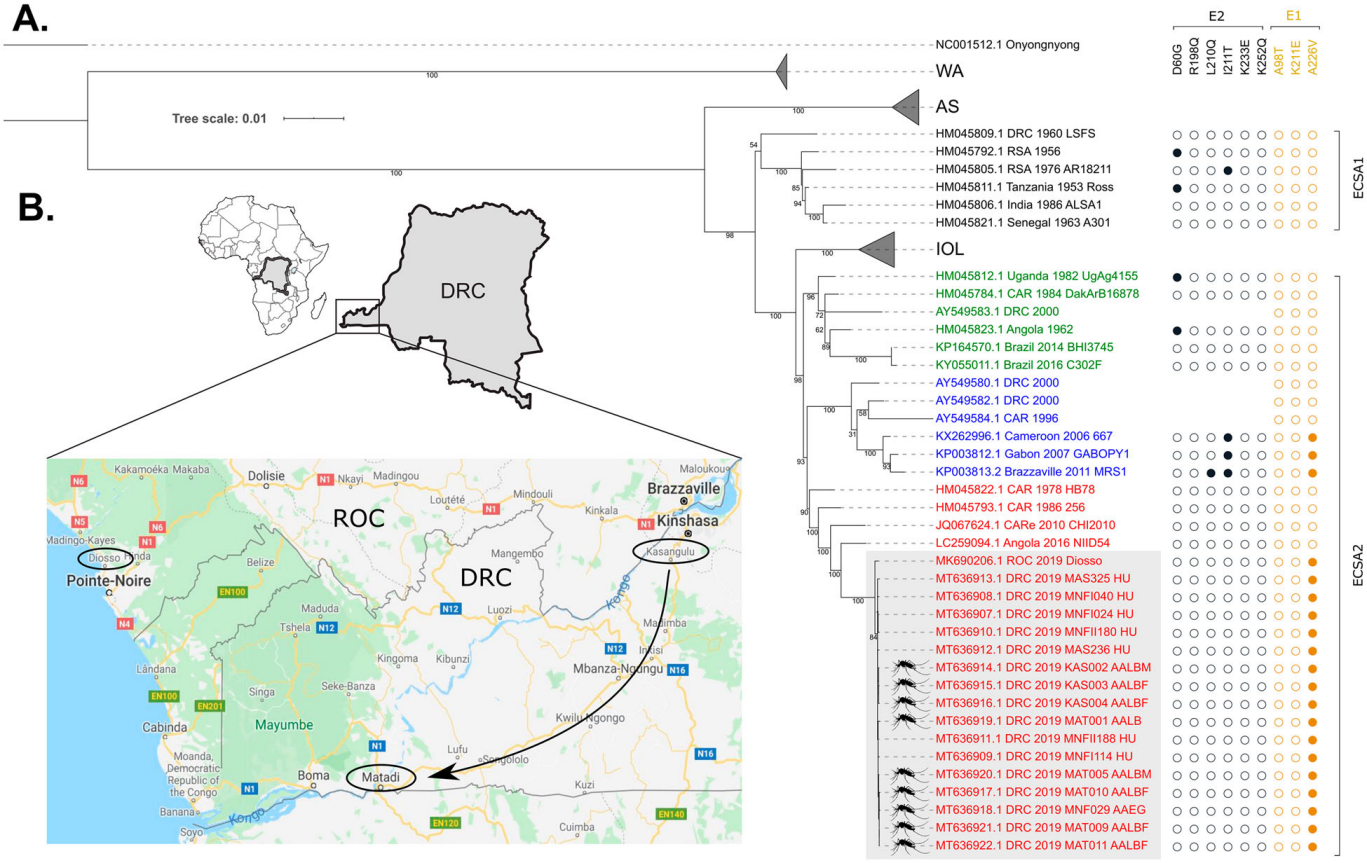
(A) Full genome maximum likelihood tree of CHIKV nucleotide sequences derived from seven patients and nine mosquitoes (labelled by a mosquito symbol) during the 2019 ROC/DRC outbreak (highlighted by the grey box) together with a panel of previously published CHIKV sequences. For DRC 2000 and CAR 1996 only the E1 sequences were available. Leaf labels contain the Genbank accession number, country, year, and name of the sequence. Next to the tree leaves, full spheres indicate the presence of a particular E1 or E2 mutation. The tree was inferred with iqTree using GTR + F+I + G4 and 100 bootstrap replicates and rooted using O’nyong-nyong virus as outgroup. The root of the tree was cropped to improve its visualization. Scale bar represents the average number of nucleotide substitutions per site. This analysis involved 122 nucleotide sequences and 11,612 positions. ECSA: East, Central, and South African; WA: West African; AS: Asian; IOL: Indian Ocean Lineage; ROC: Republic of the Congo; DRC: Democratic Republic of the Congo; CARe: Central African Region; CAR: Central African Republic; HU: human; AALB(M|F): *Aedes albopictus* (male|female); AAEG: *Aedes aegypti*; MAT: Matadi; MNF: Mont-Ngafula; MAS: Masa; KAS: Kasangulu. (B) Map of the Democratic Republic of the Congo showing the town of Diosso and the capital Brazzaville in the ROC and where samples were obtained in the DRC, i.e. the capital Kinshasa and cities Kasangulu, and Matadi. The arrow indicates the spread of CHIKV in the DRC.

We then obtained whole genome sequences (nucleotide 58 – 11,669 in HQ456251.1) from seven human and nine mosquito samples through a PCR-based approach on Oxford Nanopore's minION platform (Genbank MT636907 – MT636922). Phylogenetic analysis showed that all sequences from this outbreak formed a monophyletic group within one of the three distinct clades that can be observed in the ECSA2 lineage (coloured green, blue, red in Figure 1(A)). The most closely related sequences were imported cases in Japan from Angola in 2016 [20], and in China from Central Africa in 2010, with similarities of 98.9 and 98.7% respectively. All obtained sequences also harboured the A226V mutation (Figure 1(A)). These molecular observations correspond exactly to what was then reported about a coinciding CHIKV outbreak in the ROC [21]. However, on the nucleotide level, our sequences diverged at five synonymous positions from the circulating strain in the ROC and at one position in the 3’ UTR, with the ROC sequence being genetically closer to the ECSA reference (S Table 5) as well as its closest relatives in the phylogenetic tree.

The consensus sequences of both ROC and DRC samples also contained an 81 bp deletion (nt 11,379–11,459) comprising most of the 3’ UTR direct repeat (DR) 2a region, although viral variants without this deletion were also present at the intrasample, viral population level.

Surprisingly, all genomes obtained from this outbreak contained the epistatic E2-211 isoleucine which *in vitro* limits the emergence of A226V, supported by the fact that all of the A226V-containing ECSA-CHIKV sequences available in the ViPR database (*n*=117), showed threonine instead of isoleucine at position E2-211. When we examined the viral population, as opposed to the majority rule consensus sequence, for each of our samples at position E1-226 and position E2-211, we could not detect the alternative variants E1-226A and E2-211T at levels distinguishable from sequencing error (data not shown). Hence, these alternative variants also weren't present in the viral population as minority variants and being overshadowed by the majority variant when generating the consensus sequence.

## Discussion

In this study, we molecularly characterized a CHIKV outbreak that hit the DRC in early 2019. We were able to detect and sequence the virus in both human and mosquito samples and confirmed that the circulating virus belonged to the ECSA lineage. The virus was also phylogenetically linked to an ongoing outbreak in the ROC, neighbouring DRC, where the first suspected case presented itself on 7 January 2019 in Diosso town, 25 kilometres north of Pointe-Noire [21]. However in DRC, the first suspect cases were already reported in Kinshasa by the end of November 2018. On the 7th of January, these patients were confirmed to be positive for CHIKV IgM and IgG antibodies. From mid-February, suspect cases were reported in Matadi, DRC's main harbour town (Figure 1(B)). These timings suggest a more inland start of the outbreak, around Kinshassa and Brazzaville rather than Diosso. It is plausible that due to the similarity with malaria symptoms and the limited CHIKV diagnostic capacity, CHIKV cases remained undetected in Brazzaville late 2018. The tree topology, sampling times, and the genetically more ancestral Diosso sequence, would suggest that the virus spread from the ROC to the DRC. The viral sequence between both epidemics only differed by six synonymous nucleotide changes. Potentially these mutations are the result of a founder effect when one or very few CHIKV-infected people crossed the natural bottle neck provided by the Congo river between Brazzaville, ROC and Kinshasa, DRC.

Phylogenetic analysis further revealed that the sequences of this outbreak contained the *Ae albopictus* adaptive mutation E1-A226V, yet did not cluster with previously reported outbreaks in the DRC nor with the only other known A226V containing ECSA2 strains, obtained from Gabon (2007), Cameroon (2006), and the ROC (2011) [22]. This suggests that the E1-A226V mutation found in our analysis has been acquired independently in response to a similar selection pressure for transmission by *Ae. albopictus*. The above scenario of convergent evolution would require a substantial colonization of the area by this mosquito. This is in line with the preponderance of *Ae. albopictus* in Diosso, ROC [21] as well as with our entomological data where we found *Ae. albopictu*s more frequently than *Ae. aegypti* in two out of three affected areas.

However, the fact that E1-226V was observed for the first time in the absence of E2-211T raises questions as E2-211T was shown to be essential for the increased midgut infectivity by E1-226V. One hypothesis could be that 226V/211I provides some advantage over 226A/211I in subsequent steps of the CHIKV infection process in *Ae. albopictus.* Higher frequencies of E1-226V have been observed in viral populations in mosquito saliva as compared to the corresponding bodies, suggesting that E1-226V can be selected for at this anatomical barrier [23]. Hence this would mean that our observation represents a very early vector-host switch, where E1-226V was selected prior to E2-211T. However, at least in the three months following the first detected case, we could not observe the emergence of E2-211T in our samples from the DRC.

Another, perhaps less plausible, explanation, could be that E2-211T and E1-226V were selected for in an unreported ancestor of this outbreak which then reverted back to E2-211I. However, this requires the loss of E-211T to be at least neutral, or to be providing some kind of selective advantage. Tsetsarkin et al have suggested that E2-211I might play an important role for CHIKV maintenance in the enzootic African cycle involving non-human primates and *Ae. africanus* [10]*.* So one could speculate that E1-226V and E2-211T were selected for in an unreported, urban epidemic but were then lost during subsequent sylvatic cycling.

Interestingly we also found an 81 bp deletion in the viral genomes of this outbreak that overspans the DR 2a region in the 3’ UTR. This deletion has been observed before in a 1976 South African sequence (HM045805.1). Conserved DR regions and stem-loop structures in the 3’ UTR of arboviruses are believed to play an evolutionary role in maintaining efficient transmission in multiple hosts. Specifically, CHIKVs with deletion of different DRs have been shown to exhibit a spectrum of replication reduction in mosquito cells but not in vertebrate cells [24]. Reverse genetics experiments could reveal a potential functional effect of this deletion and/or whether it is related to the occurrence of E1-226V with E2-211I.

Of note, we found CHIKV in larvae and adult males of *Ae. albopictus* mosquitoes which suggests that vertical transmission of the virus occurs under field conditions in *Ae. albopictus*. Until now, vertical transmission of CHIKV has only been reported for *Ae. aegypti* in both laboratory and field studies and for *Ae. albopictus* in laboratory studies [25–27]. Yet the precise implication for the epidemiology and the rate at which the vertical transmission occurs, warrants further investigation.

Overall, our analysis is limited due to the lack of (historical) sequences from Africa. Furthermore, since we only sampled mosquitoes around CHIKV foci in humans, it remains possible that we undersampled areas with *Ae. aegypti.* This mosquito remains a competent vector for CHIKV regardless of the presence of E1-A226V and/or E2-211T, as is illustrated by the one positive *Ae. aegypti* pool we found. Hence, we cannot conclude that the E1-A226V mutation allowed for this outbreak to occur. Nevertheless, it's noteworthy that *Ae. albopictu*s, as an invasive species in Africa, has only been expanding in central Africa from the early 2000s [5] and that E1-A226V was detected in all four CHIKV outbreaks reported since. This suggests that CHIKV is adapting to the increased presence of *Ae. albopictus* in Africa.

## Supplementary Material

S_table_5_ROC_vs_DRC.xlsx

S_table_4_CHIKV_panel.xlsx

S_table_3_CHIKV_ECSA_scheme.xlsx

S_table_2_Sequencing_stats.xlsx

S_table_1_RT-qPCR.xlsx

## References

[1] Paixão ES, Rodrigues LC, Costa M da CN, et al. Chikungunya chronic disease: a systematic review and meta-analysis. Trans R Soc Trop Med Hyg. 2018; 112: 301–316. doi: 10.1093/trstmh/try063 30007303

[2] Pialoux G, Gaüzère BA, Jauréguiberry S, et al. Chikungunya, an epidemic arbovirosis. Lancet Infect Dis. 2007;7:319–327. doi: 10.1016/S1473-3099(07)70107-X 17448935

[3] Zeller H, Van Bortel W, Sudre B. Chikungunya: its history in Africa and Asia and its spread to new regions in 2013-2014. J Infect Dis. 2016;214:S436–S440. doi: 10.1093/infdis/jiw391 27920169

[4] Volk SM, Chen R, Tsetsarkin KA, et al. Genome-scale phylogenetic analyses of Chikungunya virus reveal independent emergences of recent epidemics and various evolutionary rates. J Virol. 2010;84:6497–6504. doi: 10.1128/JVI.01603-09 20410280 PMC2903258

[5] Kraemer MUG, Sinka ME, Duda KA, et al. The global compendium of *Aedes aegypti* and *Ae. albopictus* occurrence. Sci Data. 2015;2:1–8. doi: 10.1038/sdata.2015.35 PMC4493829 26175912

[6] Tsetsarkin KA, Vanlandingham DL, McGee CE, et al. A single mutation in Chikungunya virus affects vector specificity and epidemic potential. PLoS Pathog. 2007;3:1895–1906. doi: 10.1371/journal.ppat.0030201 PMC2134949 18069894

[7] Tsetsarkin KA, Chen R, Leal G, et al. Chikungunya virus emergence is constrained in Asia by lineage-specific adaptive landscapes. Proc Natl Acad Sci USA. 2011;108:7872–7877. doi: 10.1073/pnas.1018344108 21518887 PMC3093459

[8] Tsetsarkin KA, Chen R, Weaver SC. Interspecies transmission and Chikungunya virus emergence. Curr Opin Virol. 2016;16:143–150. doi: 10.1016/j.coviro.2016.02.007 26986235 PMC4824623

[9] Tsetsarkin KA, Weaver SC. Sequential adaptive mutations enhance efficient vector switching by Chikungunya virus and its epidemic emergence. PLoS Pathog. 2011;7:e1002412. doi: 10.1371/journal.ppat.1002412 22174678 PMC3234230

[10] Tsetsarkin KA, McGee CE, Volk SM, et al. Epistatic roles of E2 glycoprotein mutations in adaption of chikungunya virus to *Aedes albopictus* and *Ae. Aegypti* mosquitoes. PLoS One. 2009;4:e6835. doi: 10.1371/journal.pone.0006835 19718263 PMC2729410

[11] Wahid B, Ali A, Rafique S, et al. Global expansion of Chikungunya virus: mapping the 64-year history. Int J Infect Dis. 2017;58:69–76. doi: 10.1016/j.ijid.2017.03.006 28288924

[12] Pastorino B, Muyembe-Tamfum JJ, Bessaud M, et al. Epidemic resurgence of Chikungunya virus in Democratic Republic of the Congo: identification of a new Central African strain. J Med Virol. 2004;74:277–282. doi: 10.1002/jmv.20168 15332277

[13] Ido E, Ahuka S, Karhemere S, et al. Dengue virus infection during an outbreak of Chikungunya virus in Democratic Republic of Congo. Les Ann Africaines Médecine. 2016;10:2461.

[14] Proesmans S, Katshongo F, Milambu J, et al. Dengue and Chikungunya among outpatients with acute undifferentiated fever in Kinshasa, Democratic Republic of Congo: A cross-sectional study. PLoS Negl Trop Dis. 2019;13:e0007047. doi: 10.1371/journal.pntd.0007047 31487279 PMC6748445

[15] Biggerstaff B. PooledInfRate, version 4.0: a Microsoft Office add-in to compute prevalence estimates from pooled samples. Fort Collins Centers Dis Control Prev USA. 2009.

[16] Clancy A, Crowley B, Niesters H, et al. The development of a qualitative real-time RT-PCR assay for the detection of hepatitis C virus. Eur J Clin Microbiol Infect Dis. 2008;27:1177. doi: 10.1007/s10096-008-0556-9 18551325

[17] Panning M, Grywna K, Van Esbroeck M, et al. Chikungunya fever in travelers returning to Europe from the Indian Ocean region, 2006. Emerg Infect Dis. 2008;14:416–422. doi: 10.3201/eid1403.070906 18325256 PMC2570846

[18] Quick J, Grubaugh ND, Pullan ST, et al. Multiplex PCR method for MinION and Illumina sequencing of zika and other virus genomes directly from clinical samples. Nat Protoc. 2017;12:1261–1266. doi: 10.1038/nprot.2017.066 28538739 PMC5902022

[19] Appassakij H, Khuntikij P, Kemapunmanus M, et al. Viremic profiles in asymptomatic and symptomatic chikungunya fever: a blood transfusion threat? Transfusion. 2013;53:2567–2574. doi: 10.1111/j.1537-2995.2012.03960.x 23176378

[20] Takaya S, Kutsuna S, Nakayama E, et al. Chikungunya fever in traveler from Angola to Japan, 2016. Emerg Infect Dis. 2017;23:156–158. doi: 10.3201/eid2301.161395 27983938 PMC5176218

[21] Fritz AM, Taty T, Portella C, et al. Re-emergence of Chikungunya in the Republic of the Congo in 2019 associated with a possible vector-host switch. Int J Infect Dis. 2019;84:99–101. doi: 10.1016/j.ijid.2019.05.013 31096054

[22] Moyen N, Thiberville SD, Pastorino B, et al. First reported chikungunya fever outbreak in the Republic of Congo, 2011. PLoS One. 2014;9:1–21. doi: 10.1371/journal.pone.0115938 PMC4277398 25541718

[23] Stapleford KA, Coffey LL, Lay S, et al. Emergence and transmission of arbovirus evolutionary intermediates with epidemic potential. Cell Host Microbe. 2014;15:706–716. doi: 10.1016/j.chom.2014.05.008 24922573

[24] Chen R, Wang E, Tsetsarkin KA, et al. Chikungunya virus 3′ untranslated region: adaptation to mosquitoes and a population bottleneck as major evolutionary forces. PLoS Pathog. 2013;9:e1003591. doi: 10.1371/journal.ppat.1003591 24009512 PMC3757053

[25] Jain J, Kushwah RBS, Singh SS, et al. Evidence for natural vertical transmission of Chikungunya viruses in field populations of *Aedes aegypti* in Delhi and Haryana states in India—a preliminary report. Acta Trop. 2016;162:46–55. doi: 10.1016/j.actatropica.2016.06.004 27282096

[26] Chompoosri J, Thavara U, Tawatsin A, et al. Vertical transmission of Indian Ocean lineage of chikungunya virus in Aedes aegypti and Aedes albopictus mosquitoes. Parasites and Vectors. 2016;9:227. doi: 10.1186/s13071-016-1505-6 27108077 PMC4842298

[27] Ferreira-De-Lima VH, Lima-Camara TN. Natural vertical transmission of dengue virus in *Aedes aegypti* and *Aedes albopictus*: a systematic review. Parasites and Vectors. 2018;11:77. doi: 10.1186/s13071-018-2643-9 29391071 PMC5793400

